# HCV and lichen planus

**Published:** 2011-02-01

**Authors:** Alfredo Rebora

**Affiliations:** 1 C7O Clinica Dermatologica dell’Università, University of Genoa, Italy

**Keywords:** Lichen planus, HCV, Hepatitis

Dear Editor,

Mahboobi et al. should be commended for providing a clear and exhaustive review of the literature on the intriguing topic of the association of HCV infection with Lichen Planus [1]. It is somewhat surprising, however, especially for a dermatologist such as myself, to read that the histopathology of LP is nonspecific, but for cutaneous LP the specificity is out of question. Actually, the reference that Mahboobi et al. cite for this point concerns oral LP, and most of their statements refer to the oral form of LP. The point is not immaterial, as it was the striking similarity of microscopic LP features with those of the liver affected by HCV-related active hepatitis that should suggest that a connection between the two conditions does exist. In both conditions, a T-cell infiltrate impinges on the cells of the epithelial structure that are in direct contact with the corium: keratinocytes of the basal layer of the epidermis in LP and hepatocytes of the murallium of the hepatic lobe in active hepatitis. Also in both conditions, T-cells induce apoptosis of the epitheliocytes, disorganize the epithelium, and in some instances destroy it (erosive forms). Some time ago, when I described the first cases of a severe hepatic disease in patients with erosive LP [2], I was amazed not only by the striking similarity, but also by the fact that nobody had noticed it before. Certainly, not all the observations and epidemiological studies concur on the relationship between LP and HCV infection, and the geographical explanation may not be completely convincing. In addition to the explanation provided by Mahoobi et al. the possibility that OLP diagnoses might not always be correct cannot be overruled given the nonspecificity of OLP histopathology. Another epidemiological observation that cannot be neglected suggests a strict connection between LP and HCV infection. Specifically, this perspective has to do with a disease that is certainly HCV related: porphyria Cutanea Tarda (PCT). All arguments that have been made for the LP-HCV connection have been made for PCT as well. Yet, the explanation that is universally accepted is the geographical one: PCT is prevalent in the regions in which HCV infection is prevalent, and nobody contests that HCV is the major etiologic factor of PCT. Even the combination of the three diseases has been reported [3]. Certainly, HCV is not the sole cause of LP. In my view, LP is a cell-mediated immune reaction to various agents, including viruses, the most important of which are hepatotropic. In fact, LP is a relatively rare but well-recognized reaction to HBV vaccination [4]. The real problem may simply be that most studies are retrospective, which makes it difficult to establish whether HCV exposure occurs prior to or after the onset of LP. The occurrence of LP reactions after HBV vaccination irrespective of the type of vaccine used, however, strongly suggests that LP occurs after the infection. I agree with the authors that no definite conclusion can be reached at this point and also with their statement that "screening of LP patients with ELISA is cost-effective only with the presence of other risk factors". The safety of patients and of oral specialists deserves attention in hyperendemic countries, and LP, regardless of the site affected, provides an invaluable clue in this regard.
